# Supplementary material for: Netcombin: An algorithm for constructing optimal phylogenetic network from rooted triplets
Source: PLoS One. 2020 Sep 18;15(9):e0227842. doi: 10.1371/journal.pone.0227842 (PMC7500971; doi:10.1371/journal.pone.0227842)
Supplement: S4 File — (ZIP) [file pone.0227842.s004.zip › SequenceData-Triplet/sequenceData-10-20-30-0-Triplet/length 300, numseq 40, rate 10^-7/9 seq.pdf]

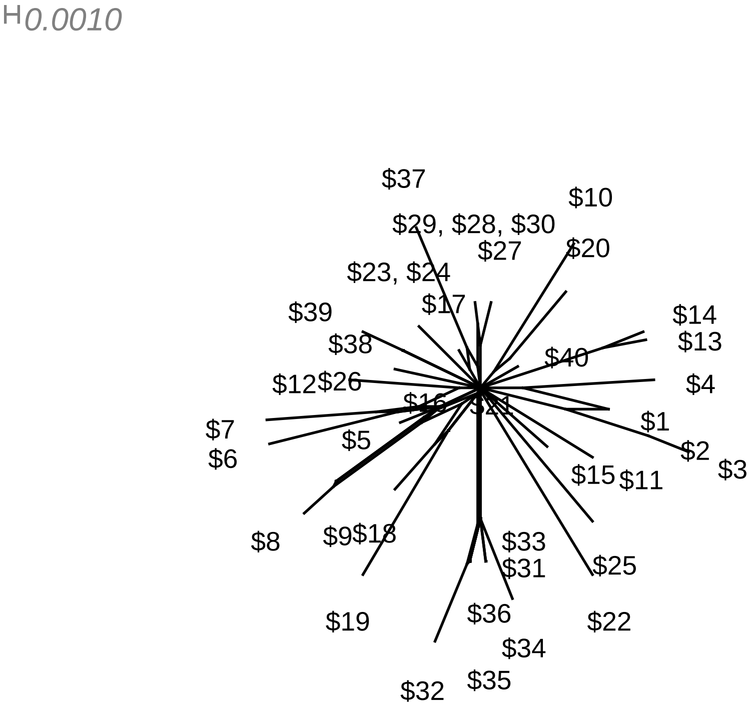
0.0010
$37
$10
$29, $28, $30
$27
$20
$23, $24
$17
$39
$14
$13
$38
$40
$12
$26
$4
$16
$21
$1
$7
$5
$2
$6
$3
$15
$11
$8
$9
$18
$33
$31
$25
$36
$19
$22
$34
$35
$32
